# Which Primates Recognize Themselves in Mirrors?

**DOI:** 10.1371/journal.pbio.1001024

**Published:** 2011-03-01

**Authors:** James R. Anderson, Gordon G. Gallup

**Affiliations:** 1 Department of Psychology, University of Stirling, Stirling, Scotland; 2 Department of Psychology, State University of New York at Albany, Albany, New York, United States of America

## Summary

Interest in the comparative study of mirror self-recognition persists because of the implications for self-awareness and the possibility of a cognitive divide among primates. Evidence from many studies carried out over 40 years shows that humans and great apes are distinguished from other nonhuman primates by their capacity for self-recognition. We review some recent developments in the field, with critical reference to claims that monkeys show self-recognition. Focusing on methodological issues, we conclude that there is no compelling evidence for mirror self-recognition in any non-ape primate species.

## Evidence for Self-Recognition

Since the original demonstration of mirror self-recognition in chimpanzees and the failure of macaques to show self-recognition [1], many studies have investigated mirror-image reactions in monkeys and apes. This literature points to the following general conclusion regarding how nonhuman primates interpret their reflection in mirrors: members of most species of great apes have shown compelling evidence that they recognize themselves, but no monkey has done so [2]. However, this apparent divide within the order Primates continues to generate resistance and considerable debate, with recurring efforts to obtain data that would bridge the divide. Here we assess recent developments in the field and focus on important methodological issues.

Great apes typically display mirror self-recognition by showing diminished social responses toward the reflection and spontaneously using the mirror to investigate parts of their body that cannot be seen without a mirror. Self-recognition is confirmed by appropriate mirror-guided exploration of otherwise unknown and invisible marks usually applied to the individual’s head [1],[3]. It is important to note the typical focus and concentration of great apes as they use the reflection to pick their teeth, explore their ears, or investigate their genitals. At best, only fleeting/incidental touches near the mark have been reported in a few monkeys during mark tests [4]. No monkey has ever been shown to use its reflection to carefully inspect a directly non-visible body part such as inside its mouth or behind an ear, in spite of repeated attempts to make things easier for monkeys. Such attempts include early and prolonged exposure to mirrors, paired and group exposure, use of angled mirrors (to avoid gaze aversion), contactable and portable mirrors, operant training of mark-directed responses, and efforts to make the mark more salient [4],[5].

Positive evidence of self-recognition in great apes and the lack of evidence in monkeys suggest the emergence of a qualitative difference in self-awareness during primate evolution, a possibility supported by other lines of evidence [2],[6],[7]. Any new claim that monkeys share the same capacity for self-recognition as great apes therefore requires rigorous evidence that must stand up to careful scrutiny.

## Monkeys’ Responses to Mirrors

One recent challenge to what’s known as the cognitive division hypothesis of self-recognition was a claim of mirror self-recognition in rhesus monkeys [8]. Individually housed monkeys sometimes manipulated an acrylic block screwed into their skull for neurophysiological experiments, and some of these manipulations occurred while the monkeys looked at their reflection in a mirror. However, the same monkeys failed to show any signs of self-recognition on a conventional mark test. Furthermore, no baseline observations were reported, and no quantitative data were presented on manipulation of the block while looking elsewhere. The mark test requires applying the mark in such a way that the subject will not know the mark is there until it is seen in a mirror. It seems likely that the acrylic blocks on the monkeys’ heads [8] provided strong tactile cues that negate their status as a suitable alternative to more conventional marks in mark tests.

One particularly intriguing aspect of how monkeys respond to mirrors is that even though they show no signs of self-recognition, they can learn to use mirrored cues to locate otherwise hidden objects [9]. In one study macaques that saw the reflection of an object suspended above them reached for the object [10]. It is therefore unsurprising that objects attached to a monkey’s head would elicit investigation. A simple test of whether implanted rhesus monkeys recognize themselves would be to remove the implants and allow the wound to heal fully. Then, submit the monkeys to a conventional mark test. If they fail, then any claim that the monkeys had learned to recognize themselves would surely be inadmissible. Contrary to the claim that traditional marks are not sufficiently salient, rhesus monkeys readily investigate such marks when applied to body parts that can be seen directly [11].

When first confronted with a mirror, monkeys typically show a range of social responses that diminish over time. Comparisons of these reactions with those shown in the presence of a live conspecific behind a transparent partition reveal some differences [12],[13]. Fish also show different brain activation in response to their mirror image and a live conspecific [14]. But simple discrimination clearly does not equate to self-recognition; although it is indeed “social,” the reflection represents atypical behavior, only ever mimicking in real time what the observer does. Therefore it is to be expected that the responses will not be exactly the same as those triggered by a live conspecific.

In contrast to great apes, however, monkeys never fully make the transition from other- to self-directed mirror-mediated responding. In one study of rhesus monkeys with years of mirror experience, the monkeys showed diminished social responses to the reflection, and mostly exhibited only passing interest in the mirror. However, simply moving the mirror to a new location (e.g., from one side of the cage to the other) produced a short-lived, but dramatic reinstatement of vigorous social responding to the reflection [15]. In a follow-up study five years later, the mirror was simply turned away from the home cage of two monkeys. Several days later when it was turned back to face the cage, both animals reacted as if they were seeing monkeys they had never seen before, showing intensive social and aggressive behavior directed toward the reflection [16].

Over the years several claims of self-recognition in monkeys have appeared, and comparative self-recognition research remains a “hot topic.” In one study, conspicuous marks were applied to the heads of cotton-top tamarins [17]. Some of the monkeys reportedly met the criteria for self-recognition, but a subsequent attempt to replicate these results failed, leading to the following conclusion: “Overall, results suggest that cotton-top tamarins fail to exhibit any evidence of mirror-guided behavior….Taken together, the results…tilt the scale back in favor of a phylogenetic gap between monkeys and at least some apes” [18].

## Diversifying Self-Recognition Research

Recent assessments of whether monkeys recognize themselves include modifications of the mark test, and alternatives to mirror-image stimulation. In one variant of the mark test, marmoset monkeys were marked with chocolate paste rather than odorless dye, in a deliberate attempt to increase the monkeys’ attention to the mark. However, this did not lead to mirror-guided exploration of the mark [19]. In an exploration of capuchin monkeys’ reactions to video images of themselves, capuchins clearly distinguished between real-time images and images delayed by one second [20]. Although they showed no explicit signs of self-recognition, they were sensitive to the visual consequences of their own movements. In another video study, Japanese macaques were extensively trained to use a tool to retrieve food, and then direct view of their hand was replaced by a video image. Bimodal sensory neurons in the parietal cortex were found to fire not only when the monkey directly saw an object approaching its hand, but also when the scene was visible only on the video monitor [21], This was interpreted as evidence that the monkeys had learned to equate the video image with their hand, but the relationship between this kind of training outcome and naturally developing self-recognition ability is unclear.

Two recent studies that involved marking the bodies of monkeys failed to find any evidence of mirror self-recognition. Adopting a procedure originally devised to assess spontaneous interest in visible marks [11], capuchin monkeys were marked on directly visible body areas to give them enhanced experience of the correspondence between their marked body and their reflection. Despite being trained to touch such marks, when marks were confined to their face the monkeys failed to use of their reflection to touch or try to remove them [22]. When pig-tailed macaques were allowed to see marks on their bodies only in a mirror, they did not use the reflection to reach for and investigate the marks. This suggests that common mechanisms underlie body and facial self-recognition [23].

In conclusion, data accumulated over decades support the original finding [1], that primate self-recognition, defined as the ability to become the object of one’s own attention, may be restricted to humans and the great apes. Although some authors prefer to consider self-awareness as a continuum [13], the weight of evidence supports the view that the ability to direct one’s attention to the self involves a qualitative cognitive shift, one that has occurred only recently in primate evolutionary history and in relatively few species.
